# Otitis media in young Aboriginal children from remote communities in Northern and Central Australia: a cross-sectional survey

**DOI:** 10.1186/1471-2431-5-27

**Published:** 2005-07-20

**Authors:** Peter S Morris, Amanda J Leach, Peter Silberberg, Gabrielle Mellon, Cate Wilson, Elizabeth Hamilton, Jemima Beissbarth

**Affiliations:** 1 Ear Health and Education Unit, Menzies School of Health Research, Darwin, Australia; 2 Institute of Advanced Studies, Charles Darwin University, Australia; 3 Northern Territory Clinical School, Flinders University, Darwin, Australia

## Abstract

**Background:**

Middle ear disease (otitis media) is common and frequently severe in Australian Aboriginal children. There have not been any recent large-scale surveys using clear definitions and a standardised middle ear assessment. The aim of the study was to determine the prevalence of middle ear disease (otitis media) in a high-risk population of young Aboriginal children from remote communities in Northern and Central Australia.

**Methods:**

709 Aboriginal children aged 6–30 months living in 29 communities from 4 health regions participated in the study between May and November 2001. Otitis media (OM) and perforation of the tympanic membrane (TM) were diagnosed by tympanometry, pneumatic otoscopy, and video-otoscopy. We used otoscopic criteria (bulging TM or recent perforation) to diagnose acute otitis media.

**Results:**

914 children were eligible to participate in the study and 709 were assessed (78%). Otitis media affected nearly all children (91%, 95%CI 88, 94). Overall prevalence estimates adjusted for clustering by community were: 10% (95%CI 8, 12) for unilateral otitis media with effusion (OME); 31% (95%CI 27, 34) for bilateral OME; 26% (95%CI 23, 30) for acute otitis media without perforation (AOM/woP); 7% (95%CI 4, 9) for AOM with perforation (AOM/wiP); 2% (95%CI 1, 3) for dry perforation; and 15% (95%CI 11, 19) for chronic suppurative otitis media (CSOM). The perforation prevalence ranged from 0–60% between communities and from 19–33% between regions. Perforations of the tympanic membrane affected 40% of children in their first 18 months of life. These were not always persistent.

**Conclusion:**

Overall, 1 in every 2 children examined had otoscopic signs consistent with suppurative ear disease and 1 in 4 children had a perforated tympanic membrane. Some of the children with intact tympanic membranes had experienced a perforation that healed before the survey. In this high-risk population, high rates of tympanic perforation were associated with high rates of bulging of the tympanic membrane.

## Background

Middle ear disease (otitis media) is common and frequently severe in Australian Aboriginal children [1,2] In the worst affected communities, perforation of the tympanic membrane (TM) may affect more than 50% of children [3,4]. Such high rates have not been described consistently in any other population in the world.

We have previously reported the early onset of otitis media with effusion (OME) within the first few weeks of life in a remote Northern Australian community [5]. In the same community, we found acute otitis media (AOM) occurred frequently, was usually asymptomatic, and predicted subsequent perforation [6,7]. Normal TM mobility was almost never seen in the first few years of life. Whether the same pattern of disease exists in other remote Aboriginal communities in our region is not clear.

There have been at least 9 surveys of ear disease in children living in Northern and Central Australia [6]. The most comprehensive survey was conducted as part of the National Trachoma and Eye Health Program in the late 1970's [8,9]. This study found that around 30% of Aboriginal children 0–9 years in Central Australia and around 20% in the rest of the Northern Territory had severe otitis media (associated with a perforated tympanic membrane). The investigators did not identify many children with OME. This may have been because their assessment did not include either tympanometry or pneumatic otoscopy.

There have not been any recent large-scale surveys using clear definitions and a standardised middle ear assessment in young Australian children living in remote Aboriginal communities. As clinicians in developed countries recognize that episodes of otitis media often do not require treatment, there is a need to identify the clinical features of otitis media associated with poor outcome. A better understanding of otitis media in populations with high rates of perforation of the TM is likely to be helpful. The primary aim of this study was to measure the prevalence of different types of otitis media in young Aboriginal children across a range of communities in Northern and Central Australia using a standardised assessment. Secondary aims included the provision of diagnostic and management training for health staff in the participating communities. The survey was conducted prior to the introduction of the 7 valent pneumococcal conjugate vaccine so the impact of this vaccine and other interventions could be assessed.

## Methods

The survey was conducted from May to November 2001. While most of the survey took place over the Australian winter, daytime temperatures are warm to hot (around 20–30°C) in the central desert and northern tropical climates. We visited in 29 remote Aboriginal communities from four different regions: Darwin Rural (6), East Arnhem (7), Katherine District (5) and Central Australia (11 communities). The Central Australian communities included 4 communities from the Nganampa health region which is located in South Australia. We approached community councils and clinics if we believed that: i) most clients were Aboriginal; ii) most children in the community used the clinic as their primary source of health care; iii) the infant immunisation program was operating effectively; and iv) medical records documenting all clinic presentations were maintained. We did not approach communities in the Barkly region. Each community received 1–2 scheduled visits. Visits were extended if a large proportion of families who were known to be in the community were not seen.

### Participants

Children were identified from the Northern Territory birth register, immunisation register, and clinic records. Children were eligible to participate in the study if they were: i) Aboriginal (self-identified); ii) aged between 6 and 30 months on the first day of the visit to their community; iii) resident in the community according to clinic staff (not including out-stations). Non-Aboriginal children (<5% of permanent residents) and children visiting the community were excluded. These remote Aboriginal communities are extremely disadvantaged by Australian standards. English is generally spoken as a second language, housing is overcrowded, and employment and educational opportunities are limited. Nearly all mothers breast feed their infants for >12 months and many smoke. Very few communities are able to offer child care services. Most houses do not have indoor heating. Exposure to outdoor fires in the dry season is frequent. Children are generally part of large extended families and have many 'siblings'. Malnutrition, lower respiratory tract infections, diarrhea, and skin infections are common illnesses and the main causes of admission to hospital. Uptake of scheduled immunisations is very good. Primary health care in all the participating communities follows the clinical practice guidelines of the Central Australian Rural Practitioners Association Standard Treatment Manual. This recommends antibiotic treatment of all episodes of acute otitis media for 5 days with either amoxicillin 40–60 mg/kg/day or cotrimoxazole 2.4/12–4/20 mg/kg/day or procaine penicillin 40–60 mg/kg/day [10]. Episodes of severe otitis media were most often associated with infection with one or more types of *Streptococcus pneumoniae *or *Haemophilus influenzae *(and frequently both). At the time of this survey, infection with penicillin resistant pneumococci and treatment failure were common [11].

### Clinical assessment

We conducted a parental questionnaire, review of clinic medical records, and a clinical assessment. The following information was recorded: date of birth, history of past ear infections, last audiological assessment, pneumococcal immunisation status, and any health problems at the time of examination. Additional OM risk factor data were not collected. All clinical assessments were made by ear health research officers. Assessments were made using a tympanometer (Grason Stadler GSI 38), a voroscope (WelchAllyn LumiView) with Siegel's speculum for pneumatic otoscopy, and a video-otoscope (WelchAllyn). Ear canals were cleaned under direct vision. Data were collected using standardised forms. Video-otoscope images were stored as ATI files using ATI TV Player Version 6.0 software. All video-otoscope images and tympanograms were later reviewed by a second trained observer. Any disagreements in the assessments were resolved by discussion with the study paediatrician.

We categorised middle ear states as follows: (1) normal; (2) otitis media with effusion (OME); (3) acute otitis media without perforation (AOM/woP); (4) AOM with perforation (AOM/wip); (5) dry perforation; and (6) chronic suppurative otitis media (CSOM). We based our criteria for diagnosis on recommendations for clinical practice in this population:[12] (i) OME – intact and non-bulging TM and Type B tympanogram; (ii) AOM/woP – any bulging of the TM and Type B tympanogram; (iii) AOM/wiP – middle ear discharge observed and perforation present for less than six weeks or covering less than 2% of the pars tensa of the tympanic membrane; (iv) dry perforation – TM perforation without any discharge observed; (v) CSOM – middle ear discharge observed and perforation present for longer than six weeks and covering at least 2% of the pars tensa of the TM.

The diagnostic criteria for AOM/woP are the most controversial. Our otoscopic criteria (middle ear effusion plus bulging of the TM) are consistent with national clinical practice guidelines [12] and previous studies in this population [4], a systematic review of international literature [13], and diagnostic studies using tympanocentesis [14-16]

The final middle ear diagnosis reflected the child's more severely affected ear. Perforation of the TM was present if the child had either AOM/wiP, dry perforation, or CSOM. We considered children with a perforation of the TM to have severe OM. We considered children with AOM/woP, AOM/wiP, or CSOM to have suppurative otitis media (presence of a pus producing infection).

### Ethical considerations

Each community was visited by an Aboriginal Liaison Officer. Community involvement was confirmed following negotiation with the local Heath Clinic and Community Council. The study was approved by the Human Research Ethics Committees of the Menzies School of Health Research and the Northern Territory Department of Health and Community Services, and the Central Australian Human Research Ethics Committee. The study was also approved by the research advisory committees of the Tiwi Health Board, Katherine West Health Board, and the Nganampa Health Council. Individual informed consent was obtained from each parent or guardian by a project officer or an Aboriginal Liaison Officer using oral, pictorial, and written explanations.

### Statistical analysis

We used Stata Version 8.0 to analyse all data [17]. The overall numbers and percentages of children with different types of otitis media were described. The 95% confidence intervals (95%CI) for these estimates were adjusted to allow for the clustering effect of living within the same community using robust standard errors. Comparisons of the prevalence of severe OM between different regions and communities were made using Fisher's exact test and Pearson's chi-squared test adjusted for the design effect of clustering by community. The cumulative probability of experiencing a perforation was estimated by using a Kaplan-Meier survival curve. The first perforation was assumed to occur on the day it was documented in the medical records or on the day of the assessment if it had not been recorded before. A p value of <0.05 was considered significant and all statistical tests were two-sided.

## Results

We initially consulted with 38 communities. 29 agreed to be in the study. Of the 9 communities who decided not to participate in the study, three did not regard ear disease in young children as a priority, five regarded ear disease as a priority and were interested in participating (but were unable to confirm approval prior to the proposed visit), and one regarded ear disease as a priority but had insufficient resources to support research.

We examined 709 of the 916 (78%) children who were eligible to participate in the study. The proportion of eligible children seen in individual communities ranged from 42% to 100%. Of the 207 eligible children who were not seen, 145 were absent from the community at the time of the visit. The other 62 children either could not be found in the community or were unable to attend the clinic as arranged. The children from the different health regions were similar in terms of age and gender (Table 1). Very few children were acutely unwell or febrile at the time of the examination. Twenty-four children (3%) were noted to have another health problem that required medical treatment (mainly skin infections). Parents usually preferred to defer the examination of sick children until they were feeling better.

**Table 1:** Baseline characteristics of children seen by region.

	**Darwin Rural**	**East Arnhem**	**Katherine District**	**Central Australia**	**All Regions**
	**No. (%)**	**No. (%)**	**No. (%)**	**No. (%)**	**No. (%)**
Total Children Listed	347	330	144	229	**1050**
Eligible Children	299 (100%)	310 (100%)	123 (100%)	184 (100%)	**916 (100%)**
Eligible Children present during study visit	270 (90%)	253 (82%)	87 (71%)	161 (88%)	**771 (84%)**
Eligible Children present during study visit and examination performed	260 (87%)	230 (74%)	83 (67%)	136 (74%)	**709 (77%)**
Mean Age (months)	18.3	15.8	16.7	19.1	**17.4**
Male	123 (48%)	126 (55%)	44 (52%)	67 (49%)	**360 (51%)**
History of AOM	216 (83%)	157 (68%)	49 (59%)	106 (78%)	**528 (76%)**
History of perforation	110 (42%)	86 (37%)	36 (43%)	70 (51%)	**302 (44%)**

Abbreviations: No. = number; % = percent; AOM= acute otitis media.

### Prevalence of middle ear disease

The ear examination was completed successfully in 698 children (98%). In 11 children, neither tympanic membrane (TM) could be seen. Nearly all children examined (91%, 95%CI 88, 94) had some form of otitis media (Table 2). Otitis media (all types) was as common in the children aged 6 to 18 months of age (352/378, 93%) as in the older children aged 18 to 30 months of age (304/331, 92%). Severe OM (TM perforation) was less common in younger children (22% versus 26%). Boys had a similar prevalence to girls for any OM (92% versus 93%) and slightly lower for severe OM (22% versus 25%). Children of parents who reported they did not have ear infection on the day of examination were much less likely to have severe OM (48/499, 10% versus 119/204, 58%). Similarly, children who did not have a previous perforation documented in their clinic notes were also much less likely to have severe OM (24/407, 6% versus 145/302, 48%). The prevalence of any OM was similar for both groups.

**Table 2:** Prevalence of the different types of otitis media in Aboriginal children aged 6–30 months from remote communities in Northern and Central Australia (95% confidence intervals adjusted for the clustering effect of living in the same community).

**Diagnosis**	**Children (N = 709)**	**Percentage (95% CI)**
Not determined	11	2% (0.4, 3)
Normal	53	8% (5, 10)
Unilateral OME	72	10% (8, 12)
Bilateral OME	219	31% (27, 34)
AOM/woP	185	26% (23, 30)
AOM/wiP	47	7% (4, 9)
Dry Perforation	15	2% (1, 3)
CSOM	107	15% (11, 19)
Any suppurative OM	339	48% (44, 52)
Any Perforation (Severe OM)	169	24% (19, 29)

Any OM	**647**	**91% (88, 94)**

Abbreviations: N = number; % = percent; CI = confidence interval; OME = otitis media with effusion; AOM/woP = acute otitis media without perforation; AOM/wiP = acute otitis media with perforation; CSOM = chronic suppurative otitis media; OM = otitis media.

Eight percent (95%CI 5, 10) of all children had 2 normal middle ears. 10% (95%CI 8, 12) had unilateral OME and 2% (95%CI 1, 3) had a dry perforation in their worst affected ear. The remaining 80% of children had either bilateral disease or a suppurative infection (AOM or CSOM). Those children with a suppurative infection were treated with antibiotics.

Bilateral OME was the most common diagnosis and affected 31% (95%CI 27, 34) of children (Table 2). Another 26% (95%CI 23, 30) of children (generally well at the time of assessment) had a bulging TM and were classified as having AOM/woP. 7% (95%CI 4, 9) had recent middle ear discharge (AOM/wiP) and 15% (95%CI 11, 19) had chronic middle ear discharge (CSOM). Overall, 24% (95%CI 19, 29) of children had a perforation of the TM and 48% (95%CI 44, 52) of children had a suppurative ear infection (ie. AOM/woP, AOM/wiP, or CSOM).

### Community and regional variation

**Figure 1:**
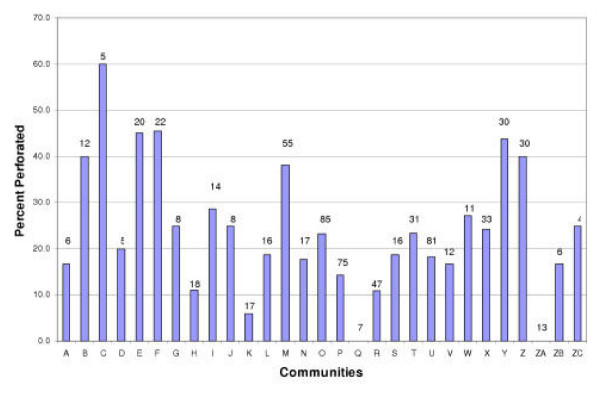
Percentage of children examined with a perforation in each of the 29 communities at the time of examination. (Number at top of bar represents the total number of children examined).

In 27 of the 29 communities, at least one child examined had a perforation in one or both tympanic membranes (Figure 1). The two communities where no child had a perforation were relatively small (community Q- 7 children examined and community ZA- 13 children examined). At the other end of the spectrum, in one community three of five of children (60%) had perforations. Since the number of children seen in individual communities was generally small, estimates of perforation prevalence at the community level lack precision. However, the range in perforation rates was substantial (0–60%). Seven communities had a perforation prevalence >35% and six communities had a perforation prevalence <15% (Figure 1). These differences in perforation prevalence between communities were unlikely to have occurred by chance (Pearson's chi-squared test adjusted for design effect, p = 0.07).

**Figure 2:**
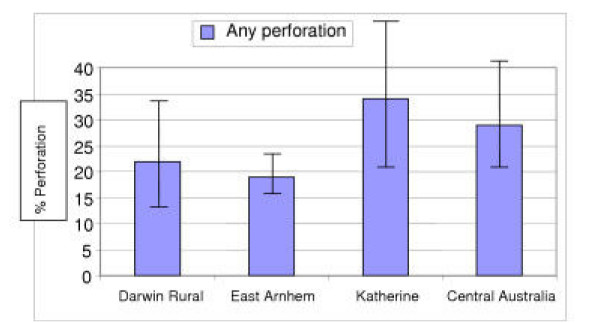
Percentage of young children from remote communities in Northern and Central Australia with severe otitis media by health region (error bars represent 95% confidence intervals adjusted for the clustering effect of living in the same community).

Perforation of the TM affected 58/260 children (22%) in Darwin Rural, 43/230 (19%) in East Arnhem, 28/83 (34%) in Katherine, and 40/136 (29%) in Central Australia (Figure 2). These regional differences were only statistically significant when you assumed that children living within communities were independent (Fisher's exact test, p = 0.015; Pearson's chi-squared test adjusted for design effect, p = 0.12). The community prevalence of severe OM varied from 0–38% in Darwin Rural, 11–27% in East Arnhem, 0–44% in Katherine, and 6–60% in Central Australia. The risk of perforation for an individual child was associated with community of residence in the Darwin Rural and Katherine regions (Fisher's exact test, p = 0.022 and p = 0.028 respectively).

### Onset of severe otitis media

**Figure 3:**
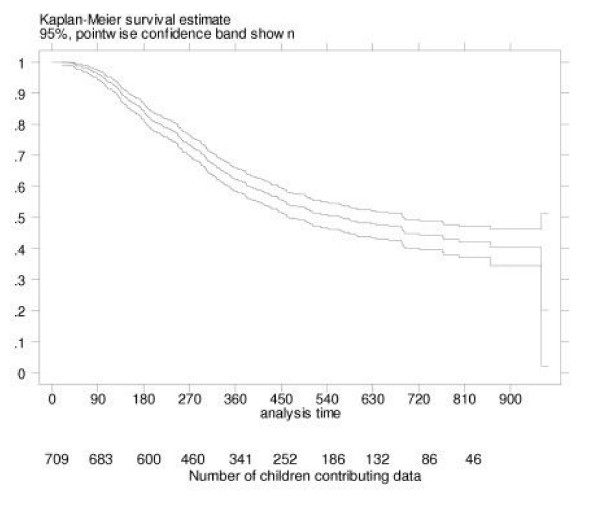
Time in days to 1^st ^perforation recorded in medical records in young Aboriginal children from 29 remote communities in Northern and Central Australia.

Perforation of the TM remains extremely common in this population. Perforations were documented in the medical records from 19 days of age (Figure 3). Most perforations were first documented in children aged 3–18 months. By 6 months of age, 14% (95%CI 12, 17) of children had a documented perforation. This increased to 31% (95%CI 27, 34) by 12 months and 40% (95%CI 36, 45) by 18 months of age. Initial perforations were less commonly documented after 18 months of age. By 24 months of age, 45% (95%CI 41, 50) of children had a documented perforation. Overall, 43% of children aged 6–30 months in this study had a perforation documented and 24% had a perforation at the time of our assessment.

## Discussion

This is the first study in over twenty years to document the middle ear state of young Aboriginal children from a range of remote communities in Northern and Central Australia. Unfortunately, the rates of OM (all types), TM perforation, and suppurative OM (AOM/woP, AOM/wiP or CSOM) remain extremely high. The impact of severe otitis media on hearing is likely to be significant and may contribute to poor educational and employment outcomes [18]. In this study, only 20% of children were likely to have normal hearing and did not require medical or audiological treatment. Importantly, there was considerable variation in the prevalence of perforation of the TM and suppurative OM between communities. Relatively few community-based surveys anywhere in the world have described a prevalence of TM perforation higher than 5% [19]. Based on our results, it would appear that infections associated with middle ear effusion begin early in life and are persistent. Children who are well at routine assessment will often have a bulging TM consistent with AOM. Initial perforations occur early in life. These perforations can heal but a substantial proportion progress to CSOM.

### Strength of evidence and applicability

This survey was conducted in communities across 4 geographically diverse regions (including the tropical north and central desert). Examinations were standardised and performed by trained observers. We used clear, mutually exclusive definitions for each diagnosis. While some children were absent from the community at the time of our visit, a more comprehensive survey would not have altered the conclusions of this study.

The rates of different types of OM are always dependent on the diagnostic criteria used. The diagnosis of OME generally requires either tympanometry or pneumatic otoscopy [20]. Tympanometry is likely to be a reliable and objective method of identifying middle ear effusion in this age group. The distinction between a bulging tympanic membrane and a neutral or retracted tympanic membrane in a young child is more difficult [13,21] The use of video-otoscopy and review of all images by a second trained observer improved the consistency of our assessments and allows for our findings to be easily verified. We are therefore confident that bulging TMs are extremely common in this population. Other studies have shown that bulging TMs are excellent predictors of AOM in children (positive likelihood ratio 51, 95% CI 36, 73) [13,22]. However, we accept that clinicians who define AOM/woP as a condition that requires the recent onset of symptoms would describe different rates of AOM/woP and OME [23,24].

The prevalence of severe OM (TM perforation) can be compared to the only other large scale survey of children in this age group [9]. Since the previous survey also found 20–30% of children in the same region had a perforated TM, it would appear that there have not been substantial improvements over the last 25 years. While other Indigenous populations (in New Zealand, USA and Canada) have reported reductions in rates of TM perforation, the reasons for this are not clear [25-27].

While we can be confident that the overall rates of TM perforation are extremely high in this population (95%CI 19 to 29%), it is more difficult to predict the perforation prevalence for other individual communities. We found substantial variation in rates of TM perforation between communities. There were no obvious reasons for this. The more disadvantaged desert communities did tend to have higher perforation rates but this was not always consistent. In all regions, some communities that appeared to be similar had very different rates of TM perforation. While severe otitis media in remote Aboriginal children is thought to be a disease of poverty, this can be difficult to establish empirically [4]. In the analysis of the only national survey of middle ear disease in Aboriginal Australians, low socioeconomic status of community (odds ratio 3.3) and inequality within community (odds ratio 1.8) were both found to be important risk factors [28]. For studies limited to remote Aboriginal communities in our region, the uniformly high levels of unemployment, the lack of educational opportunities, and childrearing within large extended families mean that an accurate assessment of the impact of poverty is difficult.

The variation in rates of TM perforation means a local survey using a standardised assessment (including tympanometry and trained observers) or the development of a perforation register would be needed to establish the extent of the problem in a community outside this study. We would strongly recommend focusing on children under 3 years of age. This is the time when: i) infections are most likely to be acute and thus responsive to treatment; ii) the impact of hearing loss associated with severe ear disease is greatest.

### Other relevant information

The problem of severe OM affecting young Aboriginal children in rural and remote communities around Australia has been appreciated for some time. In addition, suppurative otitis media is a good marker of increased risk for other suppurative respiratory infections. Aboriginal children in this region are known to have extremely high rates of rhinosinusitis, bronchitis, pneumonia, and bronchiectasis [9,29-31]. Interventions that are effective in preventing suppurative OM are likely to provide substantial benefits for respiratory health in general.

### Recommendations for research

A follow-up survey of children resident in the same communities after the introduction of the 7-valent pneumococcal conjugate vaccine is in progress. In addition to studies evaluating immunisation, we also need investigations into the impact of i) changes in hygiene practices; and ii) changes in antibiotic prescribing and compliance. These studies must focus on young children, especially those under 18 months of age. Studies assessing the accuracy of diagnosis in remote communities, and the ability of prognostic tools to predict severe OM are also appropriate. The relationship between a bulging TM and perforation needs to be described through longitudinal studies with frequent assessment in a high-risk population. Further surveys in this population should be linked to prioritisation of service delivery, improvements in the quality of health care, or well-designed intervention studies.

### Recommendations for practice

The primary health care priorities in remote communities should be to: i) support strategies that reduce the transmission of bacterial infections to infants and toddlers; ii) encourage timely immunisation; iii) advise on effective communication strategies for hearing impaired children; iv) provide frequent and accurate assessment of middle ear disease in the first 18 months of life; v) educate families about the appropriate management for different types of ear disease; and vi) help families give prolonged antibiotic treatment to their children with persistent suppurative otitis media. Evidence-based recommendations for clinical practice guidelines for otitis media in Aboriginal and Torres Strait Islander Populations are now available [12] A training video and workbook have been developed to improve the quality of diagnostic assessment in remote communities[21] However, even with perfect primary health care, rates of severe OM may remain high unless we address the extreme poverty, the paucity of educational opportunities, and high unemployment in remote Aboriginal communities. This will require substantial investment into a range of services that include health, education, housing, transportation, and recreation.

## Conclusion

In this standardised assessment of middle ear state in 29 remote Aboriginal communities, 1 in every 2 children (aged between 6 and 30 months) had otoscopic signs consistent with suppurative ear disease and 1 in 4 children had a perforated tympanic membrane. Some of the children with intact tympanic membranes had experienced a perforation that healed before the survey. In this high-risk population, high rates of tympanic perforation were associated with high rates of bulging of the tympanic membrane.

## Competing interests

Wyeth Vaccines approved the protocol and funded the study. The final decision about the content of the submitted paper was made by the authors. Wyeth Vaccines has also provided travel funding to the Ear Health and Education Unit to attend conferences on pneumococcal disease.

## Authors' contributions

All the authors contributed to the development of the study protocol and the writing of the paper. PM and AL prepared the initial funding application, supervised the protocol development and data collection, completed the analysis, and wrote the first draft of the manuscript. PS wrote the first draft of the study protocol an assisted with the data collection. GM managed the project, organized the field trips, and provided feedback of results to participating communities. GM, CW, EH collected the data. JB prepared the database and cleaned all the data. All authors read and approved the final manuscript.

## Pre-publication history

The pre-publication history for this paper can be accessed here:



## References

[B1] Couzos S, Metcalf S, Murray R (2001). Systematic review of existing evidence and primary care guidelines on the management of otitis media in Aboriginal and Torres Strait Islander populations.

[B2] Coates HL, Morris PS, Leach AJ, Couzos S (2002). Otitis media in Aboriginal children: tackling a major health problem. Med J Aust.

[B3] Watson DS, Clapin M (1992). Ear health of aboriginal primary school children in the Eastern Goldfields Region of Western Australia. Aust J Public Health.

[B4] Morris PS (1998). A systematic review of clinical research addressing the prevalence, aetiology, diagnosis, prognosis and therapy of otitis media in Australian Aboriginal children. J Paediatr Child Health.

[B5] Leach AJ, Boswell JB, Asche V, Nienhuys TG, Mathews JD (1994). Bacterial colonization of the nasopharynx predicts very early onset and persistence of otitis media in Australian aboriginal infants. Pediatr Infect Dis J.

[B6] Morris PS (1998). Improving the medical management of otitis media and other chronic bacterial respiratory diseases in rural and remote Aboriginal children: a systematic approach.

[B7] Morris PS, Yonovitz A, Leach AJ, Mathews JD, Tos M (1999). Are respiratory mucosal diseases the same in developing countries? Diagnosing acute otitis media in a high risk population. Otitis Media Today.

[B8] Moran DJ, Waterford JE, Hollows F, Jones DL (1979). Ear disease in rural Australia. Med J Aust.

[B9] The Royal Australian College of Opthalmologists (1980). National Trachoma and Eye Health Program.

[B10] Central Australian Rural Practioners Association (CARPA) (1997). CARPA Standard Treatment Manual.

[B11] Gibney KB, Morris PS, Carapetis JR, Skull SA, Smith-Vaughan HC, Stubbs E (2005). The clinical course of acute otitis media in high-risk Australian Aboriginal children: a longitudinal study. BMC Pediatr.

[B12] Morris P, Ballinger D, Leach A, Koops H, Hayhurst B, Stubbs L (2001). Recommendations for Clinical Care Guidelines on the Management of Otitis Media in Aboriginal and Torres Strait Islander Populations.

[B13] Rothman R, Owens T, Simel DL (2003). Does this child have acute otitis media?. JAMA.

[B14] Halsted C, Lepow ML, Balassanian N, Emmerich J, Wolinsky E (1968). Otitis media. Clinical observations, microbiology, and evaluation of therapy. Am J Dis Child.

[B15] Karma PH, Penttila MA, Sipila MM, Kataja MJ (1989). Otoscopic diagnosis of middle ear effusion in acute and non-acute otitis media. I. The value of different otoscopic findings. Int J Pediatr Otorhinolaryngol.

[B16] Leibovitz E, Satran R, Piglansky L, Raiz S, Press J, Leiberman A (2003). Can acute otitis media caused by Haemophilus influenzae be distinguished from that caused by Streptococcus pneumoniae?. Pediatr Infect Dis J.

[B17] StataCorp (2001). Stata Statistical Software: Release 7.0.

[B18] Leach AJ (1999). Otitis media in Australian Aboriginal children: an overview. Int J Pediatr Otorhinolaryngol.

[B19] Bluestone CD (1998). Epidemiology and pathogenesis of chronic suppurative otitis media: implications for prevention and treatment. Int J Pediatr Otorhinolaryngol.

[B20] Rosenfeld RM, Bluestone CD (1999). Evidence-Based Otitis Media.

[B21] McCallum G, Wilson C, Thomsen P, Angeles G, Leach A, Morris P (2002). The Ear Video Workbook- Trainer Version. Improving the diagnosis and treatment of otitis media in young Aboriginal children.

[B22] Paradise JL (1987). On classifying otitis media as suppurative or nonsuppurative, with a suggested clinical schema. J Pediatr.

[B23] Chan LS, Takata GS, Shekelle P, Morton SC, Mason W, Marcy SM (2001). Evidence assessment of management of acute otitis media: II. Research gaps and priorities for future research. Pediatrics.

[B24] McCormick DP (2002). Definition and diagnostic criteria for acute otitis media. Pediatrics.

[B25] Giles M, Asher I (1991). Prevalence and natural history of otitis media with perforation in Maori school children. J Laryngol Otol.

[B26] Todd-NW J, Bowman CA (1985). Otitis media at Canyon Day, Ariz. A 16-year follow-up in Apache Indians. Arch Otolaryngol.

[B27] Baxter JD (1999). Otitis media in Inuit children in the Eastern Canadian Arctic – an overview – 1968 to date. Int J Pediatr Otorhinolaryngol.

[B28] Hudson HM, Rockett IR (1984). An environmental and demographic analysis of otitis media in rural Australian aborigines. Int J Epidemiol.

[B29] Maxwell GM (1972). Chronic chest disease in Australian aboriginal children. Arch Dis Child.

[B30] Torzillo PJ, Waterford JE, Hollows FC, Jones DL (1983). Respiratory disease amongst Aborigines in the Pilbara. Int J Epidemiol.

[B31] Chang AB, Grimwood K, Mulholland EK, Torzillo PJ (2002). Bronchiectasis in indigenous children in remote Australian communities. Med J Aust.

